# Contact Investigation for Imported Case of Middle East Respiratory Syndrome, Germany

**DOI:** 10.3201/eid2004.131375

**Published:** 2014-04

**Authors:** Annicka Reuss, Annette Litterst, Christian Drosten, Michael Seilmaier, Merle Böhmer, Petra Graf, Hermann Gold, Clemens-Martin Wendtner, Arina Zanuzdana, Lars Schaade, Walter Haas, Udo Buchholz

**Affiliations:** Robert Koch Institute, Berlin, Germany (A. Reuss, M. Böhmer, A. Zanuzdana, L. Schaade, W. Haas, U. Buchholz);; Department of Health and Environment, Munich, Germany (A. Litterst, P. Graf, H. Gold);; University of Bonn Medical Centre Institute of Virology, Bonn, Germany (C. Drosten);; Hospital Schwabing, Munich, Germany (M. Seilmaier, C.-M. Wendtner);; Bavarian Health and Food Safety Authority, Oberschleißheim, Germany (M. Böhmer)

**Keywords:** MERS-CoV, Middle East respiratory syndrome, MERS, CoV, coronavirus, nCoV, coronavirus infections, viruses, Germany, respiratory infections, United Arab Emirates

## Abstract

On March 19, 2013, a patient from United Arab Emirates who had severe respiratory infection was transferred to a hospital in Germany, 11 days after symptom onset. Infection with Middle East respiratory syndrome coronavirus (MERS-CoV) was suspected on March 21 and confirmed on March 23; the patient, who had contact with an ill camel shortly before symptom onset, died on March 26. A contact investigation was initiated to identify possible person-to-person transmission and assess infection control measures. Of 83 identified contacts, 81 were available for follow-up. Ten contacts experienced mild symptoms, but test results for respiratory and serum samples were negative for MERS-CoV. Serologic testing was done for 53 (75%) of 71 nonsymptomatic contacts; all results were negative. Among contacts, the use of FFP2/FFP3 face masks during aerosol exposure was more frequent after MERS-CoV infection was suspected than before. Infection control measures may have prevented nosocomial transmission of the virus.

## Abstract 2

No evidence was found for nosocomial transmission of this coronavirus.

Middle East respiratory syndrome coronavirus (MERS-CoV) infection was initially reported to the World Health Organization (WHO) in September 2012 (*1*,*2*). By November 11, 2013, a total of 153 laboratory-confirmed cases of human infection with MERS-CoV had been identified; 64 (42%) of those with confirmed cases had died (*3*). Most (63%) case-patients had severe respiratory disease; 76% also had >1 underlying chronic condition (*4*). The median age of case-patients was 50 years (range 14 months to 94 years). All cases were directly or indirectly related to countries in the Middle East or on the Arabian Peninsula.

MERS-CoV shows a close genetic relationship with coronaviruses found in bats (*1*,*5*–*10*), but no zoonotic link has been confirmed. Person-to-person transmission has been reported in the work environment, among family contacts, or to health care workers (HCWs) (*11*–*13*). Although situations involving consecutive human transmission events have been documented (*13*), none of the known clusters have led to sustained person-to-person transmission in the general population. In Europe, single imported infections have been reported in the United Kingdom, Germany, France, and Italy, and secondary cases have been reported in the United Kingdom, France, and Italy (*12*,*14*,*15*). Because a large proportion of cases are fatal and the virus could acquire the ability to spread more efficiently (as was the case with severe acute respiratory syndrome coronavirus), WHO has recommended thorough contact investigations for confirmed human cases to identify, quantify, and prevent person-to-person transmission (*16*).

In Germany, MERS-CoV infection was initially reported in a person from Qatar (*17*). He was in his third week of illness and was already on mechanical ventilation when he was admitted to a hospital in Essen in October 2012. A retrospective contact investigation found no indication of person-to-person transmission to contacts in Germany (*17*).

On March 23, 2013, the Institute for Virology of the University of Bonn reported an imported case of MERS-CoV infection to the Department of Health and Environment in Munich (City Health Department). A 73-year-old man from Abu Dhabi, United Arab Emirates, had been admitted to a hospital in Munich and had positive test results for MERS-CoV infection (Figure 1). Clinical details and virologic findings have been reported elsewhere (*18*). Briefly, the patient had underlying multiple myeloma and had received several modes of treatment, including high-dose chemotherapy and autologous stem-cell transplantation in 2009. On March 8, 2013, influenza-like illness with fever and cough developed in the patient. After his symptoms worsened, he was hospitalized in his country on March 10 with a diagnosis of pneumonia; he was intubated on March 17 and transferred by flight ambulance services to Germany on March 19, eleven days after illness onset, for further intensive care treatment and mechanical ventilation.

**Figure 1:**
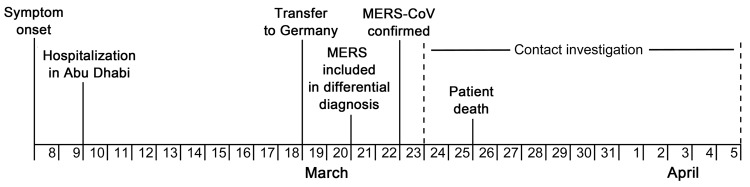
Timeline for patient history and contact investigation in imported case of Middle East respiratory syndrome (MERS), Germany, 2013.

General infection control guidelines of the Munich hospital required that patients from areas such as the Middle East, where prevalence of multidrug-resistant pathogens is high, be isolated until colonization or infection with a multidrug-resistant pathogen is ruled out. This rule is particularly enforced when patients have been previously hospitalized in the country of origin. Thus, at the time of hospital admission in Germany, the patient was isolated from other patients. When MERS-CoV infection was suspected and included in the differential diagnosis on March 21, standard hygiene measures for HCWs were changed to infection control measures as recommended for severe acute respiratory syndrome patients, including the use of FFP2 face masks for usual patient care (*19*).

MERS-CoV infection was diagnosed in the patient on March 23; he died on March 26 of multiorgan failure and acute respiratory distress syndrome. After MERS-CoV infection was diagnosed, the City Health Department, in cooperation with the state health department, the Institute for Virology in Bonn, and the Robert Koch Institute, initiated an investigation to 1) monitor all contacts of the patient to identify possible person-to-person transmission, 2) assess infection control measures, and 3) explore possible sources for the patient’s infection to prevent further cases.

## Methods

### Contact Investigation

For the investigation, the City Health Department assessed all contact persons (contacts) retrospectively and monitored them prospectively. All contacts received a questionnaire for retrospective documentation and prospective daily self-monitoring of symptoms, exposure to the patient, and infection control measures applied. For every day from March 19 through April 5, information was collected about the contacts’ distance from the patient (<2 meters vs. >2 meters); type of contact with the patient (aerosol-producing procedures, non–aerosol-producing procedures, care of patient, handling of urine catheter, handling of respiratory samples in the laboratory, handling of urine samples in the laboratory); type of protection used (surgical mask, FFP1 mask, FFP2 mask, FFP3 mask, gown, gloves, protective glasses); and symptoms experienced by the contacts (cough, fever, temperature, sore throat, diarrhea, shortness of breath). An aerosol-producing procedure was defined as respiratory suction, bronchoalveolar lavage, intubation, or bronchoscopy.

On the basis of the self-reported information in the questionnaires and personal interviews with the contacts, we divided contacts into 2 groups. Close-distance contacts had face-to-face contact with the patient (<2 meters from the patient) or direct contact with secretions or body fluids of the patient, irrespective of protective measures worn. All other contacts were classified as less-close-distance contacts. According to WHO recommendations on the duration of follow-up at that time, close-distance contacts were asked to contact the City Health Department daily for 10 days after the last exposure to the patient. Those who failed to do so were contacted by the City Health Department, supported by the occupational health service of the hospital. Less-close-distance contacts were asked to report to the City Health Department only in case of onset of symptoms.

Respiratory illnesses in contacts that occurred 1–10 days after exposure to the patient were assessed through the City Health Department by telephone contact with the contact; a respiratory tract sample was taken from any contact with respiratory illness. In addition, attempts were made to obtain paired serologic samples from all contacts, the first taken immediately after contact and the second >28 days after the last exposure.

### Patient Questionnaire

Because the MERS-CoV patient was on mechanical ventilation and could not be interviewed, family contacts who had accompanied him to Germany were interviewed about the onset of his symptoms and possible exposures in the 10 days before disease onset. For the interview, a structured questionnaire was used, and information collected was documented on paper.

### Laboratory Methods

PCR testing and serologic testing were done as described (*17*,*20*). Serum samples from contacts were tested for MERS-CoV antibodies if a serum sample was taken >28 days after last exposure. In addition, serum samples were tested for antibodies against influenza A, B, and C; rhinovirus A, B, and C; parainfluenzavirus 1, 2, 3, and 4; respiratory syncytial virus A and B; human metapneumovirus; coronavirus 229E, NL63, OC43, and HKU1; and adenovirus. All samples were analyzed at the Institute for Virology of the University of Bonn.

### Data Analysis

Data from the City Health Department’s contact monitoring, the contacts’ questionnaires, and the laboratory findings were integrated in 1 database. Results were validated and analyzed by using Stata version 12.0 (StataCorp, College Station, TX, USA).

## Results

### Contact Investigation

The City Health Department identified 83 contacts. Of these, 69 (83%) were classified as close-distance contacts and 14 (17%) as less-close-distance contacts (Table). Four (5%) of the contacts were members of the patient’s family, 16 (19%) were physicians, 25 (30%) were nursing staff, 20 (24%) were laboratory personnel, and 18 (22%) were part of other professional groups. Clinical follow-up was available for 81 (98%) contacts.

**Table:** Results of contact investigation of patient with imported MERS-CoV infection, Germany, 2013*

Data category	No. (%) contacts†	No. (%) close-distance contacts, n = 69‡	No. (%) less-close- distance contacts, n = 14‡	p value§
Contacts, n = 83				0.103
Physicians	16 (19)	11 (69)	5 (31)	
Nursing staff	25 (30)	24 (1)	1 (4)	
Laboratory personnel	20 (24)	17 (85)	3 (15)	
Family members	4 (5)	4 (100)	0	
Other	18 (22)	13 (72)	5 (28)	
Response to questionnaire	61 (73)	55 (90)	6 (10)	0.004
Aerosol exposure	15 (18)	15 (100)	0	0.054
Symptoms				0.006
Symptomatic	10 (12)	9 (90)	1 (10)	
Nonsymptomatic¶	71 (86)	60 (85)	11 (15)	
Unknown	2 (2)	0	2 (100)	
Swab samples, symptomatic contacts, n = 10				0.725
Swab sample collected	9 (90)	8 (89)	1 (11)	
No swab sample collected	1 (10)	1 (100)	0	
PCR results for symptomatic contacts with swab samples, n = 9				NA
MERS-CoV positive	0	NA	NA	
MERS-CoV negative	9 (100)	8 (89)	1 (11)	
HCoV-NL63 positive	1 (11)	1 (100)	0	
Rhinovirus positive	2 (22)	2 (100)	0	
Serologic test results				0.007
MERS-CoV positive	0	NA	NA	
MERS-CoV negative	60 (72)	54 (90)	6 (10)	
Not done	23 (28)	15 (65)	8 (35)	
Serologic testing among symptomatic contacts, n = 10				0.107
MERS-CoV positive	0	NA	NA	
MERS-CoV negative	7 (70)	7 (100)	0	
Not done	3 (30)	2 (67)	1 (33)	
Serologic testing among nonsymptomatic¶ contacts, n = 71				0.095
MERS-CoV positive	0	NA	NA	
MERS-CoV negative	53 (75)	47 (89)	6 (11)	
Not done	18 (25)	13 (72)	5 (28)	

*Definitions of close-distance and less-close-distance contacts provided in article text. MERS-CoV, Middle East respiratory syndrome coronavirus; NA, not applicable. †Percentages are of contacts (N = 83) unless otherwise indicated. ‡Percentages are of category total. §Probability that the distribution as indicated occurs by chance given the column and row totals. ¶Nonsymptomatic contacts are asymptomatic persons and those who were symptomatic before exposure.

A respiratory symptom or fever developed in 10 (12%) contacts. Of these, swab specimens were collected from 9 (90%) and blood samples from 7 (70%). All 9 swab specimens were negative for MERS-CoV; 1 (11%) was positive for CoV NL-63, and 2 (22%) were positive for rhinovirus. All 7 serum samples were negative for MERS-CoV antibodies. All symptomatic contacts had >1 sample type (respiratory swab or serum) collected for laboratory testing; results of PCR and serologic testing were available from 6 (60%), PCR only from 3 (30%), and serologic testing only from 1 (10%). In addition, serologic test results were available for 53 (75%) of the 71 nonsymptomatic contacts; all were negative for MERS-CoV antibodies. Overall, persons for whom serologic testing results were available were more likely to be close-distance contacts than were persons without available serologic results (p = 0.007; Table).

The 4 family members who accompanied the patient were his wife, daughter, son, and son-in-law. Their ages were 35–37 years, and none reported symptoms. The patient’s children and son-in-law had their last contact with the patient on March 20 and his wife on March 23. Because no protection measures had been used until after March 20, the family members were considered at high risk for infection. All 4 provided respiratory swab and serum samples on March 24; all samples had negative results. Serum samples taken >28 days after last exposure to the patient were not available.

**Figure 2:**
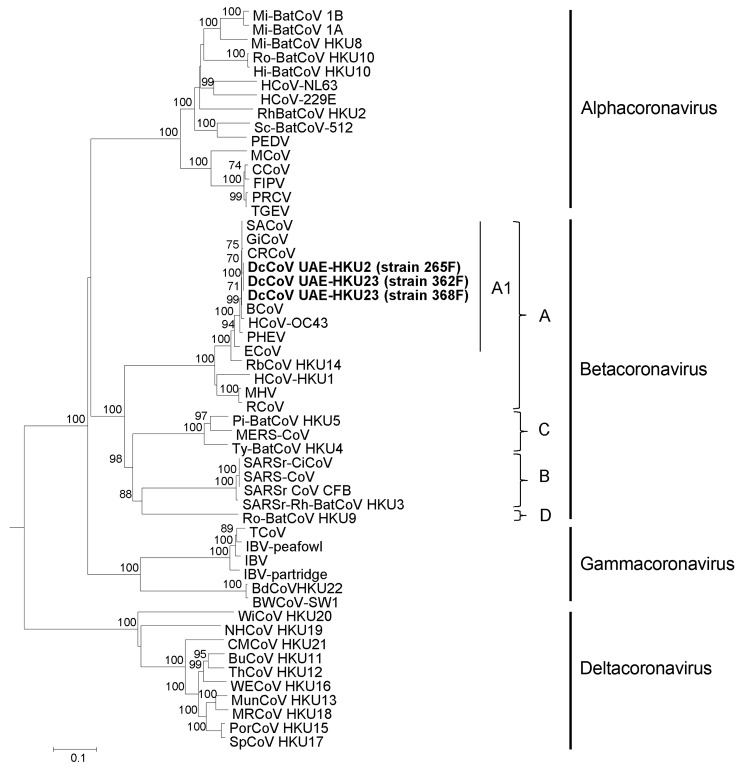
Daily number of health care workers who had contact with a patient infected with Middle East respiratory syndrome (MERS) coronavirus who was hospitalized in Germany, March 19–26, 2013.

MERS-CoV infection was added to the differential diagnosis for the patient on March 21. The daily numbers of HCWs who had any contact with him (regardless of protection measures) and of those who had aerosol exposure were lower after that date than before (Figure 2): 4.4 HCW per illness day vs. 7.5 HCW per illness day (p = 0.05) and 2.8 HCW per illness day vs. 6 HCW per illness day (p = 0.03). Among HCWs with aerosol exposure, 1 (8%) of 12 daily exposures occurred while FFP2 or FFP3 masks were being used before March 21; after that date, 11 (79%) of 14 daily exposures occurred while FFP2 or FFP3 masks were being used (p<0.01).

### Patient Questionnaire

The patient was a 73-year-old married man from Abu Dhabi, United Arab Emirates; he had a medical history of multiple myeloma. At the time of his MERS-CoV infection, he was receiving corticosteroid therapy. His profession was camel breeding; in the 2 weeks before his onset of illness, 1 of his camels was reported to have had a respiratory illness. In the questionnaire, we did not differentiate between dromedary (*Camelus dromedaries*) and Bactrian (*C. bactrianus*) camels. His neighborhood had palm trees, and bats were known to dwell in the area. The patient had no known contact with other MERS-CoV patients, had no personal contacts in Qatar or Jordan, and had no travel history in the 10 days before illness onset. He consumed different types of fruit juices and cooked goat meat, beef, and sheep meat, but no raw meat. He ate dates from his region, but he reportedly did not consume date or palm syrup. Other than the camels on his farm, he had no contact with animals; he did not practice falconry and did not visit camel racetracks or animal markets.

## Discussion

We describe the case and contact investigation of a confirmed case of MERS-CoV infection that was imported to Germany. We did not identify person-to-person transmission from the patient to any of the contacts. As with the previous imported case in this country, the patient was already on mechanical ventilation when he was transferred to Germany. However, whereas the previous case was in the late third week of illness, this patient was in the second week of illness. Sample from this patient taken from different body locations and at different times were positive for MERS-CoV by PCR, and the viral load detected was several logs higher than in samples from the patient with the previous imported case (*18*,*20*). These results indicate that this patient may have been more infectious than the previous patient.

Nosocomial transmission from MERS patients to HCWs has been documented (*13*,*21*,*22*). In our study, the patient was isolated during the first 2 days of his hospital stay (before MERS was suspected), although the reason for this intervention was the hospital’s policy to isolate every patient from the Middle East, irrespective of the assumed diagnosis, because of perceived increased risk of carrying drug-resistant pathogens, rather than any special measures taken because of the patient’s respiratory illness. After MERS was suspected, HCWs used FFP2 masks significantly more frequently than they had before, and fewer HCWs had daily contact with the patient. Our result suggest that, in the later stages of this disease, the combination of standard protection measures (use of surgical masks for potentially aerosol-generating procedures), cautious handling of the patient (because of his potential to harbor drug-resistant bacteria), and possible decreased infectiousness compared with the first week of illness may have prevented transmission to HCWs. These findings also underline the importance of following WHO recommendations on infection prevention and control when managing a patient who may be infected with a pathogen that could lead to nosocomial transmission (*23*).

Regarding possible sources of infection, an extensive interview was conducted with family members because the patient could not be interviewed. The patient’s illness was likely a primary case, and possible exposures that might have caused the MERS-CoV infection were explored. Of note were the presence of bats in the neighborhood of his residence, the patient’s profession as camel breeder, and his contact with a camel that was reported to have had a respiratory illness before his own illness onset. Bats are a likely reservoir for MERS-related CoV (*5*,*8*), and serum samples from Omani racing camels have shown to have neutralizing antibodies against MERS-CoV (*24*). These findings suggest these animals’ possible relevance (e.g., as intermediate hosts) for human acquisition of MERS-CoV.

Two complementary monitoring instruments for contact persons were used: active follow-up with daily telephone contact and a self-administered monitoring questionnaire. Both methods have merits, and a combination of both is likely to ensure the most thorough contact follow-up. Advantages of personal interviews on the telephone are immediacy and the possibility for the interviewer to receive intangible information, such as the self-assessment of symptoms, as well as the opportunity to answer questions from the contacts. This process enables a more specific way to judge a person’s health status. On the other hand, a daily monitoring questionnaire provides detail in clinical information, exposure, and protection measures that might be used for more in-depth analyses (e.g., when a few contacts have become infected). Such a questionnaire could be expanded to include a section for contact persons to fill in the names of persons with whom they had face-to-face contact during each day. This information might become crucial for second-generation contact tracing when contacts under observation become infected. Rapid availability of this type of information is essential for efficient investigation of clusters or outbreaks similar to those that have been reported already (*13*).

In conclusion, we conducted a contact investigation of an imported case of MERS-CoV infection in Germany. Laboratory testing of symptomatic and asymptomatic contacts of the index case-patient did not indicate transmission of the virus. Furthermore, we documented the change from standard hygiene to infection control measures after MERS-CoV was suspected, an adaptation that may have prevented nosocomial transmission. Exposure to camels as a possible etiologic mechanism for human MERS-CoV infection requires further evidence from other studies.
